# Bilateral Total Hip and Unilateral Knee Arthroplasties in a Young Adult with Arthropathy-Associated Intestinal Epithelial Dysplasia (Tufting Enteropathy)

**DOI:** 10.1155/2018/4630759

**Published:** 2018-10-30

**Authors:** Colin Ng, Kurt Magri, Ryan Giordmaina, Duncan Whitwell

**Affiliations:** ^1^Department of Trauma and Orthopaedics, Mater Dei Hospital, Triq Dun Karm, MSD 2090 Msida, Malta; ^2^Department of General Medicine, Mater Dei Hospital, Triq Dun Karm, MSD 2090 Msida, Malta; ^3^Consultant Orthopaedic Tumour and Reconstructive Surgeon, Nuffield Orthopaedic Hospital, Oxford, UK

## Abstract

Intestinal epithelial dysplasia (tufting enteropathy) is an uncommon congenital disorder. Furthermore, its association with chronic inflammatory arthropathy is rarely documented in the literature. Low prevalence rates of 1 in 100,000 live births in Western Europe exist, with higher rates in North Africa and Middle Eastern countries. Malta, being a small Mediterranean island at the cusp between Europe and North Africa, has an anecdotal sevenfold prevalence rate. This is the first documented case report of a patient with both intestinal epithelial dysplasia and severe bilateral hip and knee arthropathy that required simultaneous bilateral hip followed by, after a short interval, unilateral knee arthroplasties. Our aim is to highlight the rapid progression of associated arthropathy as well as the successful treatment with joint arthroplasties in such extreme cases. Surgical treatment may be a necessity despite best medical efforts to halt the disease.

## 1. Introduction

Intestinal epithelial dysplasia (IED), also known as tufting enteropathy (TE), is a rare congenital disorder of the small bowel and a cause of infantile intractable diarrhoea and intestinal failure [1–3]. It was initially reported in the mid-1970s, but the pathogenesis was then unknown [4]. Only two previous reports with IED causing inflammatory arthritis have been previously documented in the literature, with the development of multiple joint chronic inflammatory arthritis and tenosynovitis [5, 6]. This is the first documented case report of a patient with this same destructive combination that underwent successful surgical correction with bilateral hip and knee arthroplasties.

Histologically, the small bowel demonstrates variable degrees of villous atrophy, epithelial crowding, epithelial disorganization, basement membrane abnormalities, and crypt hyperplasia [1, 3]. IED derives its name from the epithelial cell dysplasia which manifests as focal epithelial tufts. Tufts may affect up to 70% of villi and are not limited to the small intestine, also involving the colonic mucosa [1, 3].

The estimated prevalence of IED is around 1 in 50,000–100,000 live births in Western Europe [1]. Prevalence tends to be greater in countries where there is a high degree of consanguinity. Of note, IED is more frequent in patients of Turkish, Middle-Eastern, and North African origin. Malta, a Mediterranean European island in the Mediterranean Sea south of Sicily, reports a relatively high prevalence of this disease, albeit with what appears to be a milder phenotype [1, 6, 7]. The disorder is thought to have an autosomal recessive mode of transmission. Exonal and intronal mutations in the EPCAM gene have been identified as the responsible defects in patients with IED [7–9].

Recent case reports document a potentially fatal syndrome of IED together with choanal atresia. There may be associated morphological abnormalities (short stature, broad nasal bridge, micrognathia), ophthalmological abnormalities (chronic corneal inflammation), and haematological disorders (episodic cytopenia) [10–12].

A rare but known complication of IED is inflammatory joint arthropathy that potentially leads to permanent erosive joint changes. This leads to a spectrum of focal joint changes causing gait disorders requiring surgical interventions to complete loss of mobility needing joint arthroplasty as a last resort.

## 2. Case Report

A 21-year-old female of Maltese ethnicity, without a family history of IED, was diagnosed with IED following a Ladd procedure for intestinal malrotation at the age of three months. This was confirmed on open jejunal biopsies. Her medical treatment thus pursued shortly after this period with total parenteral nutrition (TPN) and oral and intravenous steroids. The latter had accounted for her short stature. Despite this, she led a normal life and was independently mobile and pain free up until the age of 18. She remained independently mobile until the age of 19 when she developed bilateral hip and knee arthritis.

**Figure 1:**
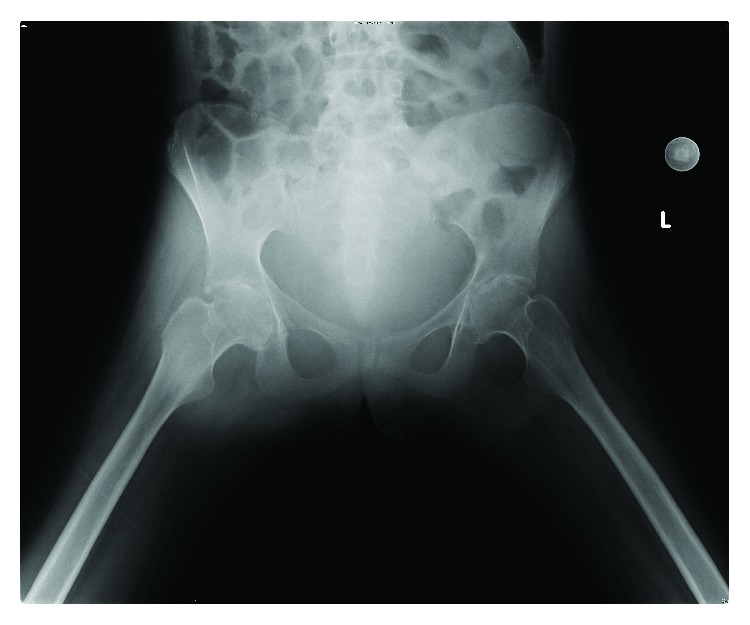
Plain hip radiographs at age 19.

**Figure 2:**
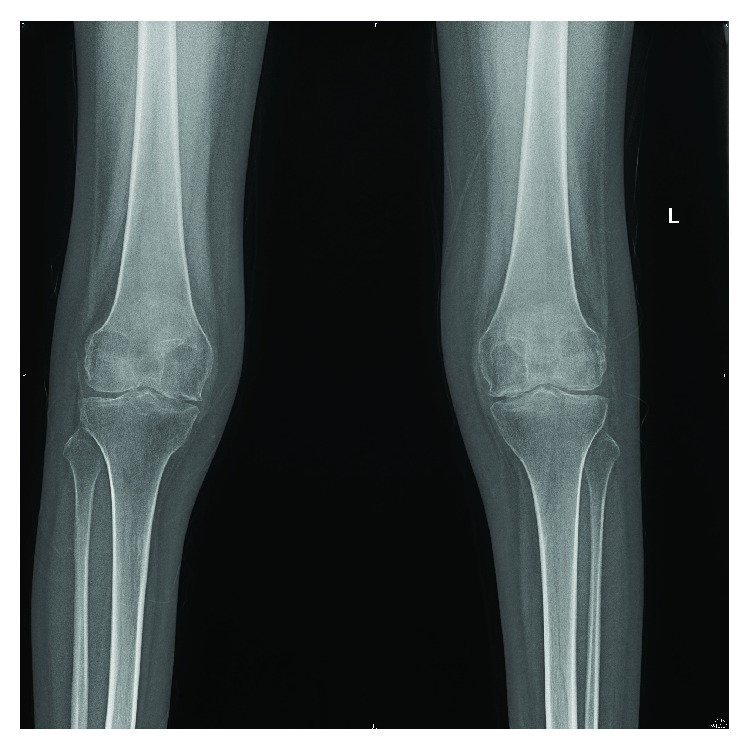
Plain knee radiographs at age 19.

**Figure 3:**
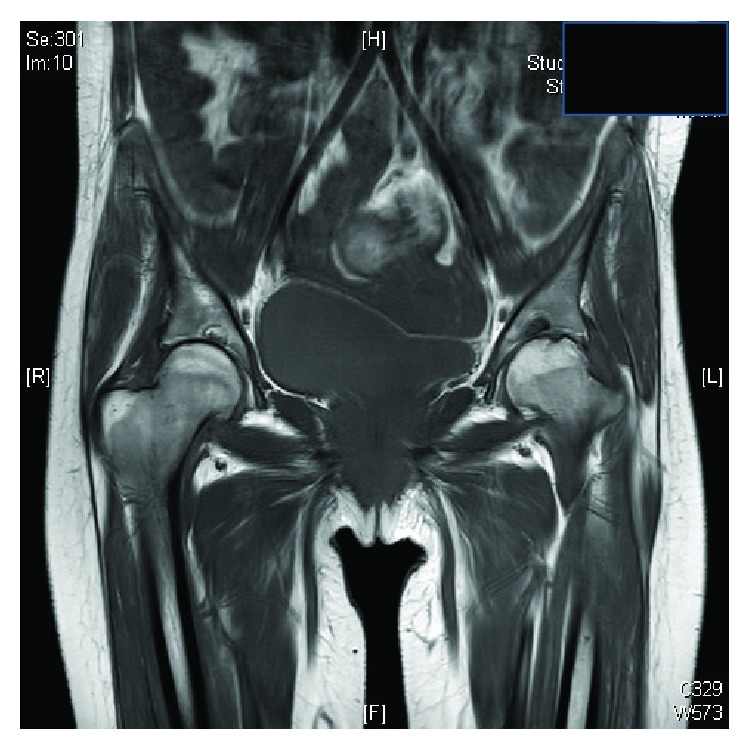
MRI of the hip at age 19. Appearances are in keeping with inflammatory arthropathy.

**Figure 4:**
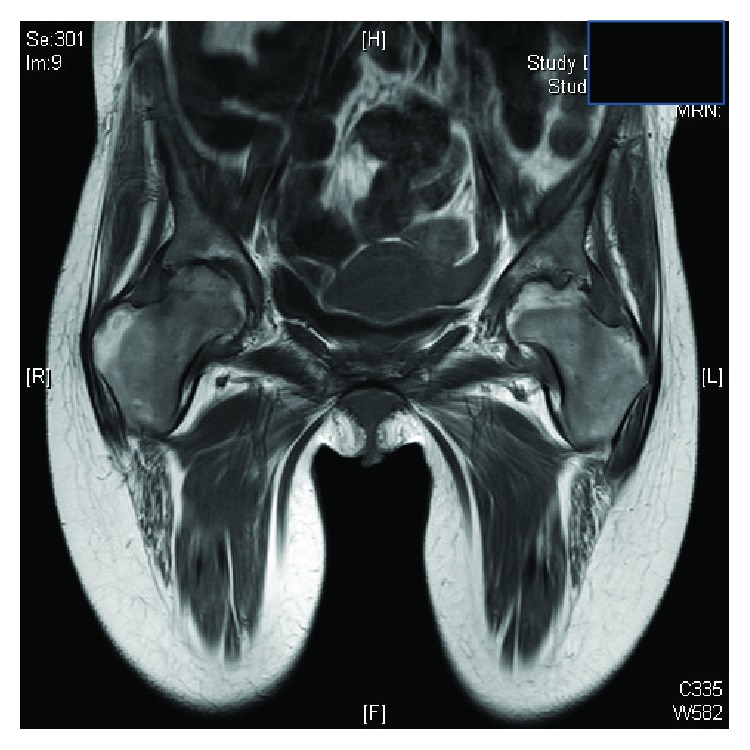
MRI of the hips at age 20.

Clinically, features were consistent with acute inflammatory polyarthropathy which were confirmed on plain radiographs (Figures 1 and 2) and serial MRIs (Figures 3 and 4). Her initial physical examination revealed marked knee effusions. Blood investigations included inflammatory markers—erythrocyte sedimentation rate (ESR) and C-reactive protein (CRP), blood count, liver function, rheumatoid factor, antinuclear antibody (ANA), and anticyclic citrullinated peptide (anti-CCP). All results were within the normal accepted range values.

In view of clinical and radiological evidence of synovitis, she was treated with methotrexate followed by infliximab. Despite such treatment, as well as several pulses of intravenous steroids, the disease progressed rapidly within six months by which time her hips were almost fused in fixed flexion/abduction and her knees were fixed in 30-degree flexion.

Initial MRI of the hips (at age 19) showed bilateral symmetrical concentric loss of joint space with areas of full-thickness chondral loss and associated subchondral cystic change in relation to either hip joint. There are small associated hip joint effusions. Overall appearances would point towards a low-grade inflammatory arthropathy, rather than primary degenerative changes.

Follow-up MRI on the hips (age 20) showed bilateral established hip articular degenerative changes with associated hip joint effusions and synovitis. No avascular necrosis pattern was being demonstrated, with bilateral femoral head small focal erosive changes.

**Figure 5:**
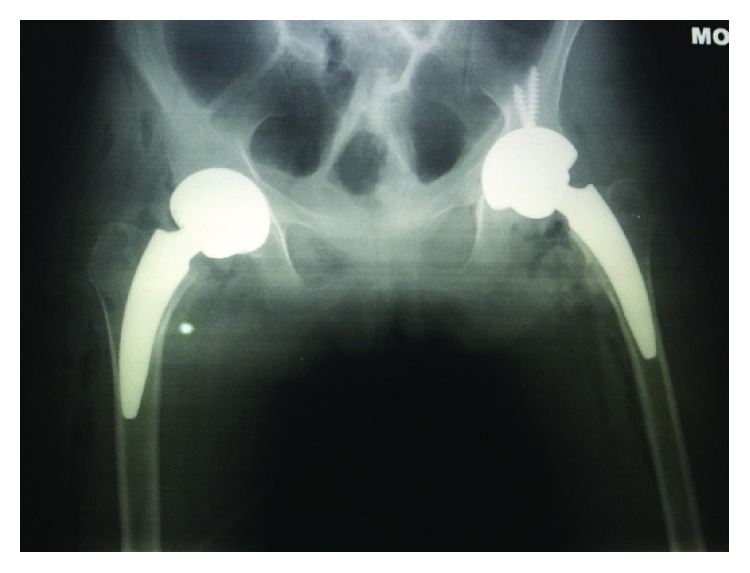
Postoperative plain radiograph of bipolar short-stemmed total hip replacements.

**Figure 6:**
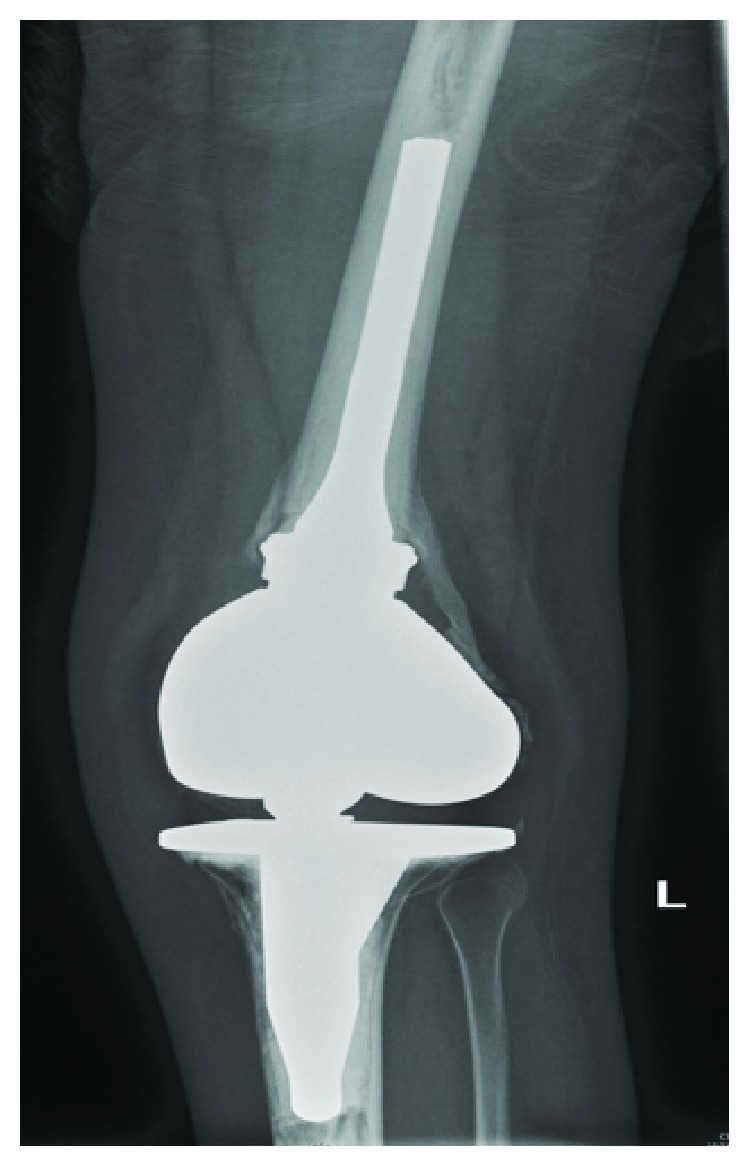
Postoperative plain radiograph of constrained left knee replacement.

**Figure 7:**
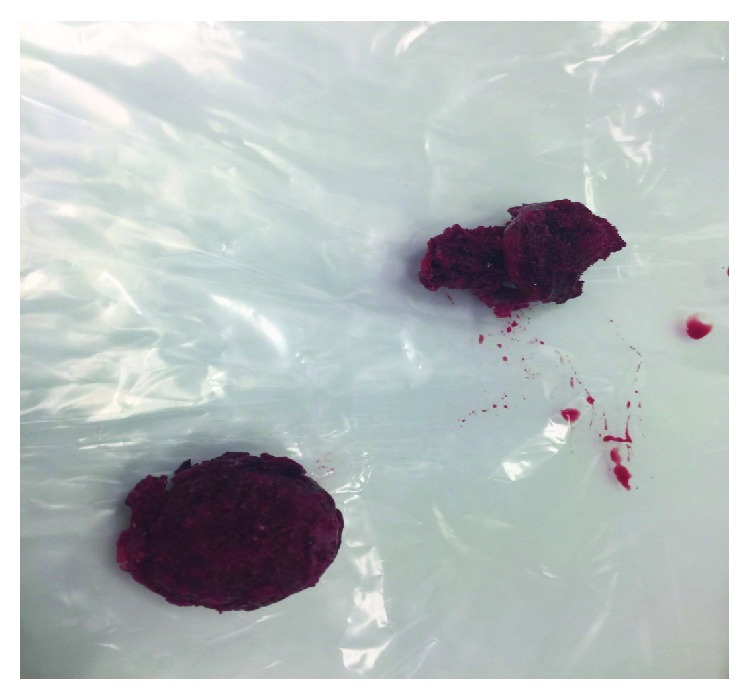
Necrotic femoral heads perioperatively.

She was referred to the Nuffield Hospital Orthopaedic Centre in Oxford, UK, where she underwent simultaneous hip arthroplasties initially (Figure 5), followed by a unilateral knee arthroplasty (Figure 6) one month proceeding. Her bone quality was found to be osteopenic more so at the distal femur compared to around the proximal femur and acetabulum. Histology of the femoral heads (Figure 7) was confirmed as inflammatory arthropathy with late degenerative changes.

## 3. Surgical Procedure

In view of her young age, the dedicated short-stem bone-preserving uncemented hip and acetabular prosthesis Corin MiniHip™ with ceramic-on-ceramic bearing was used for her bilateral hip arthroplasties (Figure 5). An anterolateral hip approach was performed in a sidelying position. The MiniHip™ has a 130-degree CCD neck angle and is a titanium femoral stem coated with a layer of hydroxyapatite with a polished distal section. Her left acetabular component was reinforced with 2 acetabular screws.

Regarding her knees, conventional total knee arthroplasty prosthesis was planned to be used; however, in view of her osteopenia, a constrained knee prosthesis, the Zimmer® distal femurs, was used instead (Figure 6).

## 4. Follow-Up

She had a prolonged but routine postoperative follow-up in Oxford, along with rigorous physiotherapy rehabilitation whereby she progressed from being bed bound to being mobile with a forearm gutter frame with assistance. Her rehabilitation continued when she was transferred back to Malta. She was followed up regularly: one, three, six, twelve, and eighteen months. She was near pain free after her twelve-month follow-up. There were no surgical-specific complications such as hip dislocations or deep or superficial joint infections. She will be continually followed up over the years and has been counselled on possible future revision arthroplasties.

## 5. Discussion

This is the first case report documented in the current literature of orthopaedic surgical treatment joint replacement for TE. It raises awareness of the probable link between IED and inflammatory arthropathy and highlights the potential rapid progression leading to end-stage arthritis. The natural history of IED rarely causes inflammatory arthropathy as a complication. However, we present a case confirmed by histopathology of femoral heads, whereby treatment with the currently known immunosuppressive agents (methotrexate, IV methylprednisolone, and infliximab) was ineffective. This is also reflected in the current literature [1, 12]. There was clinical suspicion that her deterioration was a consequence of femoral head avascular necrosis; however, radiological features and histopathology results were negative. Clinically worrying was her rapid deterioration in pain control and mobility that warranted urgent arthroplasties as an initial and final choice.

Since the first descriptions of IED by Davidson et al. [4], little has been added to the literature landscape. Even less has been documented about the severe erosive complications as a natural mode of progression of this condition.

More importantly, we hypothesize that possible underlying genetic defects may explain why some patients with TE progress to severe and progressive arthropathy whilst other patients do not show any signs of arthropathy at all. This case represents an extreme complication for a rare disease with a successful outcome thus far.
